# Gut Microbiota-Related Cellular and Molecular Mechanisms in the Progression of Nonalcoholic Fatty Liver Disease

**DOI:** 10.3390/cells10102634

**Published:** 2021-10-02

**Authors:** Eunju Park, Jin-Ju Jeong, Sung-Min Won, Satya Priya Sharma, Yoseph Asmelash Gebru, Raja Ganesan, Haripriya Gupta, Ki Tae Suk, Dong Joon Kim

**Affiliations:** Institute for Liver and Digestive Diseases, Hallym University, Chuncheon 24253, Korea; epark312@hallym.ac.kr (E.P.); jj_jeong@hallym.ac.kr (J.-J.J.); lionbanana@hallym.ac.kr (S.-M.W.); satyapriya83@gmail.com (S.P.S.); yoseph@hallym.ac.kr (Y.A.G.); vraja.ganesan@gmail.com (R.G.); haripriya.gupta@hallym.ac.kr (H.G.); ktsuk@hallym.ac.kr (K.T.S.)

**Keywords:** nonalcoholic fatty liver disease, gut microbiota, dysbiosis, gut microbial metabolites

## Abstract

Nonalcoholic fatty liver disease (NAFLD) is one of the most common and increasing liver diseases worldwide. NAFLD is a term that involves a variety of conditions such as fatty liver, steatohepatitis, or fibrosis. Gut microbiota and its products have been extensively studied because of a close relation between NAFLD and microbiota in pathogenesis. In the progression of NAFLD, various microbiota-related molecular and cellular mechanisms, including dysbiosis, leaky bowel, endotoxin, bile acids enterohepatic circulation, metabolites, or alcohol-producing microbiota, are involved. Currently, diagnosis and treatment techniques using these mechanisms are being developed. In this review, we will introduce the microbiota-related mechanisms in the progression of NAFLD and future directions will be discussed.

## 1. Introduction

Nonalcoholic fatty liver disease (NAFLD) is one of the most important causes of liver disease worldwide and the global prevalence is approximately 25%, ranging from 14–32% [1]. The prevalence of NAFLD is significantly increased in patients with other pre-existing manifestations of metabolic syndrome, such as type 2 diabetes, hyperlipidemia and hypertension [2]. While NAFLD is generally asymptomatic, NAFLD patients are at increased risk for developing other manifestations of metabolic syndrome and accompanying complications such as cardiovascular diseases [3]. NAFLD can also occur in lean patients with a normal body mass index without abdominal obesity and its prevalence is rapidly rising [4]. In addition, NAFLD and its associated manifestations have been linked to elevated insulin resistance and an increased oxidized low-density lipoprotein (LDL) to high-density lipoprotein (HDL) ratio [4,5].

NAFLD is defined as fat accumulation in the liver of patients who do not consume excessive alcohol. NAFLD has multiple stages, each of which has distinct characteristics [6]. The first stage is steatosis, which is the deposition of fat in the liver. Steatosis is considered to be reversible and a benign condition in most individuals. However, for some individuals, steatosis progresses to non-alcoholic steatohepatitis (NASH), which features inflammation in the liver and damage to hepatocytes. A subset of NASH patients develop cirrhosis and eventually hepatocellular carcinoma (HCC).

Many preclinical and clinical studies have accumulated evidence that communication between the gut microbiota, its metabolites and the liver plays a crucial role in the pathogenesis of NAFLD. The gut microbiota and the liver crosstalk extensively through the portal vein, biliary tract and systemic circulation for homeostasis [6,7]. The liver communicates with the gut by releasing bile acids and systemic bioactive mediators, including inflammatory cytokines, through the biliary tract and systemic circulation. In the gut, the host and microbiota metabolize bile acids, amino acids and monosaccharides from the diet, reaching the liver through the portal vein and are taken up by hepatocytes. In patients with NAFLD, such equilibrium between the gut and liver functions is impaired by the disturbance of gut microbiota composition [6,7]. This altered composition, known as dysbiosis, leads to increased intestinal permeability, which allows the translocation of microbes, microbial products (microbial or pathogen-associated molecular patterns) and toxins into the liver from the intestine through the portal vein, causing liver damage [8].

In this review, we summarize the effects of the gut microbiota on the progression of NAFLD, focusing on the cellular and molecular mechanisms by which gut microbiota-derived metabolites contribute to the progression of NAFLD.

## 2. Gut Microbiota and Nonalcoholic Fatty Liver Disease

The gut microbiota plays significant roles in carbohydrate digestion, bile acid metabolism, maintenance of gut barrier integrity against pathogen infection and vitamin synthesis, ultimately affecting host health and suggesting that the gut microbiome can be considered a metabolic organ in the host. The human gut microbiota is dominated by four phyla—*Bacteroidetes*, *Firmicutes*, *Proteobacteria* and *Actinobacteria*—Which represent almost 90% of the gut microbial community [9]. Of these, two phyla, *Bacteroidetes* and *Firmicutes*, are the most abundant and dominate in the intestinal tract [10]. However, changes in gut microbial composition are associated with patients with NAFLD. Roy et al. found that fecal microbiota transplantation from mice with NAFLD into wild-type mice caused NAFLD [11]. Furthermore, the abundance of specific taxa has also been associated with NAFLD in both animal and human models. More gram-negative and fewer gram-positive bacteria dominate in the gut of NAFLD patients compared to healthy controls and an increase in *Bacteroidetes* and a decrease in *Firmicutes* are associated with disease progression [12]. Compared to NAFLD patients without NASH, NASH patients harbor abundant *Bacteroides* and less *Prevotella* in fecal samples [13]. Since a plant-rich diet with *Prevotella* and a high-fat diet with *Bacteroides* are known to be associated, alterations in diet can cause an imbalance in the composition of the two genera, influencing NAFLD progression [14].

Several studies have proposed mechanisms by which alterations in the gut microbiota contribute to NAFLD. The contributions of bacterial overgrowth, increased intestinal permeability and elevated serum levels of lipopolysaccharide (LPS) have been demonstrated in NAFLD patients [15,16,17]. Overgrowth of intestinal gram-negative bacteria in NAFLD patients increases LPS production and serum levels compared to dysmetabolic patients without NAFLD [18,19]. Disruption of intestinal integrity increases intestinal permeability, which leads to bacterial translocation, and bacterial endotoxins penetrate the portal vein, which increases the risk of NAFLD development through the activation of hepatic inflammatory cells [17,20]. Bacterial endotoxins are recognized by Toll-like receptors (TLRs) on hepatocytes, which recognize several components of microbes and initiate immune responses [15]. When bacterial LPS signals through TLR4, it ultimately activates nuclear factor-kappa B (NF-κB) and proinflammatory cytokines [15,21]. Moreover, dysbiosis is associated with reduced synthesis and secretion of angiopoietin-related protein 4 (ANGPTL4), which results in increased activity of lipoprotein lipase and the accumulation of triglycerides in the liver [22,23].

## 3. Gut Microbiota-Derived Mechanism in Nonalcoholic Fatty Liver Disease

In this review, gut microbiota-derived metabolites are grouped into five classes and the mechanisms by which each class of the metabolites contributes to NAFLD pathogenesis will be discussed throughout animal and human studies.

### 3.1. Gut Microbiota-Derived Choline and Tryptophan Metabolites

Choline is an essential nutrient that is obtained through the diet and from biosynthesis in the body and is an essential component of cell membrane phospholipids [24]. Choline is involved in biological processes in the liver, including lipid metabolism, signaling through lipid second messengers and enterohepatic circulation of bile and cholesterol [24]. Studies have shown that choline-deficient diets may result in obesity and hyperglycemia and are associated with NASH caused by prevention of the synthesis and secretion of very-low-density lipoprotein (VLDL), thus leading to the accumulation of hepatic triglycerides and liver steatosis [25,26,27,28].

The gut microbiota can actively metabolize choline to trimethylamine (TMA), which is further metabolized to trimethylamine-N-oxide (TMAO) by flavin-containing monooxygenase in the liver [29,30]. Romano et al. demonstrated that conversion of choline into TMA by gut microbiota decreases choline bioavailability for the host and mimics a choline-deficient state, leading to metabolic disorders [31]. The authors developed two gnotobiotic mouse groups with a simplified gut microbiota: one group with wild-type choline-utilizing *Escherichia coli* and the other group with noncholine-utilizing *E. coli*, which failed in the conversion of choline into TMA due to knockout of a choline TMA-lyase (*cutC*) gene. Both groups were kept on a high-fat diet supplemented with 1% choline for several weeks. Using this model, Romano et al. found that choline-utilizing bacteria compete with the host for choline, modulate the gut microbiota composition and ultimately alter the host metabolome, increasing susceptibility to high-fat diet-induced metabolic disease [31]. In a clinical study, changes in the levels of choline intake in 15 female subjects altered the abundance of choline-utilizing gut bacteria, *Gammaproteobacteria* and *Erysipelotrichi*, which, along with the amount of liver fat and a single-nucleotide polymorphism associated with host choline metabolism, accurately predicted the degree to which subjects developed fatty liver when on a choline-deficient diet [32]. Patients with NAFLD are associated with high levels of TMAO in the blood [33]. Although the direct mechanism(s) by which TMA contributes to NAFLD development is not well understood, Gao et al. proposed evidence that in mice fed a high-fat diet, TMAO modulates glucose metabolism and increases insulin resistance [34]. Furthermore, TMAO causes inflammation in adipose tissue, inducing insulin resistance by increasing the serum inflammatory cytokine C-C motif chemokine 2 (CCL2) level [34].

Tryptophan is an essential nutrient derived from the diet and can be catabolized by gut microbiota to produce metabolites, mainly indole and its derivatives. As different bacteria encode different catalytic enzyme tryptophanases, different indole derivatives are produced from different bacteria: indole-3-acetic acid from *Clostridium bartlettii*; indole-3-acrylic acid from *Peptostreptococcus* spp.; indole-3-aldehyde from *Lactobacillus* spp.; indole-3-propionate from *C. adaveris*, *C. botulinum*, *C. sporogenes* and *Peptostreptococcus anaerobius*; and tryptamine from *C. sporogenes* and *Ruminococcus gnavus* [35,36,37].

In a recent cohort study of 137 subjects, the circulating levels of indole were significantly lower in obese subjects than in lean subjects [38]. The difference was inversely correlated with body mass index, liver fat accumulation and homeostasis model assessment of insulin resistance (HOMA-IR), indicating increased insulin resistance [38]. To understand the underlying mechanism, the group further conducted an animal study in which mice were fed a high-fat diet for 12 weeks and were treated with indole for the last 4 weeks. In this model, the group found that indole treatment significantly alleviated hepatic steatosis and inflammation via the master regulatory gene of glycolysis, 6-phosphofructo-2-kinase/fructose-2,6-biphosphatase 3 (PFKFB3) [38]. Indole-3-propionic acid treatment has also been shown to suppress hepatic inflammation liver injury, further attenuating high-fat diet-induced NASH [39]. This resulted from inhibition of NF-κB signaling and the reduction of proinflammatory cytokine levels in macrophages [39]. In addition to indole and indole-3-propionic acid, Krishnan et al. found that indole-3-acetic acid and tryptamine, which were depleted in high-fat diet-fed mice, decreased LPS-induced proinflammatory cytokine production in macrophages and chemokine-dependent infiltration of immune cells [40]. Additionally, in lipid-loaded hepatocytes, indole-3-acetic acid attenuated inflammatory responses and decreased the expression of fatty acid synthase (FAS) and sterol regulatory element-binding protein 1c (SREBP1c) in an aryl hydrocarbon receptor (AhR)-dependent manner [40]. In addition to suppressing hepatic inflammation, indole and indole-3-propionic acid alleviate NAFLD progression by improving intestinal barrier function [39,41,42]. Both indole and indole-3-propionic acid induce tight junction protein expression; in particular, indole-3-propionic acid regulates intestinal barrier function by activating the xenobiotic sensor pregnane X receptor (PXR), which further inhibits the TLR4 signaling pathway [39,42].

In summary, gut microbiota-derived choline and tryptophan metabolites impact NAFLD pathogenesis, in part by regulating the host metabolome, hepatic inflammation and gut barrier function through multiple host signaling pathways (Table 1). Further studies are needed to broaden our appreciation of the mechanisms of choline and tryptophan metabolites and their roles in the progression of NAFLD.

### 3.2. Microbiota-Related Short-Chain Fatty Acids and Nonalcoholic Fatty Liver Disease

Short-chain fatty acids (SCFAs) are major products generated in the gastrointestinal tract by microbial carbohydrate fermentation from indigestible carbohydrates and are mainly composed of acetate, butyrate and propionate [43]. Acetate and propionate are produced mostly by *Bacteroidetes*, while butyrate is generated by *Firmicutes* [44,45]. Following their production, SCFAs are rapidly absorbed in the colon primarily through passive diffusion, monocarboxylate transporters, or exchange with bicarbonate via solute carrier family 26 member 3 (SLC26A3), whereas their intestinal signaling effects are mediated by the activation of G protein-coupled receptors (GPCRs), which are highly expressed in small and large intestines [46,47,48,49,50,51]. Absorbed SCFAs then enter the tricarboxylic acid cycle to generate ATP and energy [52]. SCFAs that are not metabolized are released into the liver through the portal vein, where they contribute to energy metabolism [52,53].

The involvement of SCFAs in NAFLD development may be derived from their association with fatty acid synthesis and gluconeogenesis. Gut dysbiosis generally results in increased levels of SCFAs in the intestine with an increased transport of monosaccharides to the liver. Increased acetate, which is a substrate for fatty acid synthesis, in the liver causes the accumulation of triglycerides and elevated propionate in the liver promotes gluconeogenesis, contributing to weight gain [54,55]. According to Turnbaugh et al., the cecal content of obese mice featured increased acetate and butyrate levels [56]. Similar results were also observed by Schwiertz et al., who found that the total fecal SCFA concentration in obese individuals was 20% higher than that in lean individuals [57]. In a clinical study, Rau et al. reported that elevated fecal levels of SCFAs are also associated with immune cell changes in NAFLD progression [58]. Comparing NAFLD patients and healthy controls, NAFLD patients showed high levels of fecal acetate and propionate compared to healthy controls, along with an increased abundance of SCFA-producing bacteria [58]. Interestingly, this increased fecal SCFA level and altered microbiota composition were associated with a decrease in resting regulatory T cells and a higher T helper 17 cell to resting regulatory T cell ratio in peripheral blood, which are immunological features of NASH [58,59]. Elevated levels of SCFAs can also contribute to NAFLD by decreasing intestinal mobility. Activation of GPCRs, G-protein coupled receptor 41 (GPR41) and G-protein coupled receptor 43 (GPR43), by SCFAs stimulates secretion of peptide-YY (PYY), which normally inhibits gut motility and decreases the intestinal transit rate. GPCR activation by SCFAs further enhances the harvest of SCFAs from the diet and nutrient absorption and promotes hepatic lipogenesis [60].

However, several studies have shown that supplementation with SCFAs plays a protective role against NAFLD by reducing hepatic fat accumulation, hepatic inflammation and cholesterol synthesis. According to Zhai et al., supplementation of butyrate to high-fat diet-fed mice changed the composition of the gut microbiome [61]. It increased SCFA-producing bacteria but decreased endotoxin-secreting bacteria, which regulated fecal SCFA and endotoxin levels as well as decreased the expression of proinflammatory cytokines [61]. Deng et al. also found that supplementation with acetate, butyrate, or propionate reduced triglyceride and cholesterol levels in the liver and proinflammatory cytokine levels in methionine- and choline-deficient diet-fed mice [62].

These beneficial effects of SCFAs in the prevention of NAFLD can be explained by several mechanisms. First, there is evidence that SCFAs increase hepatic lipid oxidation by the AMP-activated protein kinase (AMPK)-acetyl-CoA carboxylase (ACC) pathway, which induces fatty acid oxidation in the liver and decreases hepatic fatty acid activity [63,64,65,66]. Second, SCFAs are also known to regulate hepatic lipid metabolism through glucagon-like peptide-1 (GLP-1) receptor signaling. Hepatic GLP-1 receptor expression is markedly reduced in NAFLD patients and high-fat diet-fed animals, but butyrate supplementation increases the expression of the GLP-1 receptor and further reduces liver steatosis [67,68]. In addition, activation of GLP-1 receptor increases fatty acid β-oxidation by activating the intracellular kinase activity of protein kinase A (PKA), phosphatidylinositol 3-kinase (PI3K) and AMPK and enhances insulin sensitivity by reducing c-Jun N-terminal kinase (JNK) phosphorylation and upregulating insulin receptor (IR) and insulin receptor substrate 1 (IRS1) expression [67,68]. Third, SCFAs can protect the gut barrier. Butyrate promotes the development of the intestinal barrier by facilitating the assembly of tight junctions via AMPK activation and attenuates high-fat diet-induced steatohepatitis by restoring gut microbiota dysbiosis [69,70]. Furthermore, SCFAs can inhibit NAFLD development at the epigenetic level. This will be discussed in detail in a later section of this review.

In summary, preclinical and clinical studies have shown contradictory effects of SCFAs on NAFLD progression (Table 2). Accordingly, further investigations are necessary to better understand the complexity of the interactions between gut microbiota, SCFAs and the host metabolome to identify the roles of SCFAs in NAFLD development.

### 3.3. Bile Acids and Nonalcoholic Fatty Liver Disease

The human liver synthesizes two primary bile acids, cholic acid (CA) and chenodeoxycholic acid (CDCA), from cholesterol predominantly via the rate-limiting enzyme cytochrome P450 7A1 (CYP7A1) [71]. Most primary bile acids are subsequently conjugated with amino acids, either glycine or taurine. Conjugated primary bile acids are secreted into bile for release into the intestine. Approximately 95% of the primary bile acids absorbed in the intestine return to the liver via the portal vein in a highly efficient process called enterohepatic circulation. A small fraction of the conjugated primary bile acids reach the large intestine and are converted to secondary bile acids, deoxycholic acid and lithocholic acid, by gut microbiota.

In addition to cholesterol metabolism, bile acids are involved in maintaining hepatic glucose, lipid and energy homeostasis by activating signaling pathways mainly through farnesoid X receptor (FXR) and G-protein-coupled bile acid receptor 1 (GPBAR1) [72,73]. FXR activation stimulates the expression and activation of peroxisome proliferator-activated receptor α (PPARα) to induce fibroblast growth factor 21 (FGF21) expression and secretion [74,75]. FGF21 enhances glucose uptake in adipocytes by activating mammalian target of rapamycin complex 1 (mTORC1) via mitogen-activated protein kinase (MAPK) and promotes fatty acid oxidation in adipose tissue by regulating the activity of PPARγ, which is a master transcriptional regulator of adipogenesis [76,77]. Stimulation of the GPBAR1 signaling pathway by bile acids modulates energy expenditure and regulates inflammation in the liver. Thomas et al. reported that GPBAR1 activation by its agonist INT-777 promotes intestinal secretion of GLP-1, increasing insulin sensitivity in obese mice and reducing hepatic steatosis and adiposity [78]. Shi et al. showed that in both humans and mice with NASH, hepatic GPBAR1 expression was reduced and GPBAR1-deficient mice exhibited exacerbated liver damage, increased levels of proinflammatory cytokines and facilitated macrophage polarization by promoting NACHT, LRR and PYD domain-containing protein 3 (NLRP3) inflammasome activation [79].

Clinical studies have shown that NAFLD patients have an altered systemic bile acid composition. In patients with NASH, serum levels of total and conjugated bile acid are markedly increased compared to healthy controls [80,81,82]. Furthermore, an increase in plasma levels of key bile acids is associated with higher grades of steatosis (taurocholate), lobular (glycocholate) and portal inflammation (taurolithocholate) and hepatocyte ballooning (taurocholate) [83]. According to preclinical and clinical studies, the gut microbiota has direct effects on bile acid pool size, composition and concentrations, contributing to NAFLD progression [84]. In NAFLD patients with advanced fibrosis, serum glycocholic acid and fecal deoxycholic acid concentrations were elevated compared to those in non-NAFLD controls and this high bile acid concentration was correlated with the abundance of *Bacteroidaceae* and *Lachnospiraceae* [85]. Gut microbiota-mediated changes in bile acid composition occur through interactions of bile acids with FXR and GPBAR1, inhibiting CYP7A1 expression. According to Parséus et al., conventional *Fxr*-deficient mice fed a high-fat diet showed significant changes in bile acid composition and the gut microbiota promoted weight gain and hepatic steatosis via FXR-dependent mechanisms [86]. FXR activation by bile acids in hepatocytes induces the expression of short heterodimer partner 1 (SHP-1) to suppress liver receptor homolog 1 (LRH-1)-mediated CYP7A1 expression [87]. Activation of FXR also induces the expression of FGF15 in mice and FGF19 in humans, which in turn binds to FGF receptor 4 (FGFR4) and klotho beta (KLB) in the liver to suppress CYP7A1 expression in hepatocytes in a JNK-dependent manner [88]. In patients with NAFLD, Jiao et al. found that despite the elevated levels of serum primary and secondary bile acids, FXR-mediated and FGFR4-mediated signaling was suppressed by showing increased expression levels of CYP7A1 and FGFR4. Furthermore, *Escherichia* and *Bilophila*, which are taurine- and glycine-metabolizing bacteria, showed increased abundance compared to healthy control subjects, indicating an increase in secondary bile acid production in the gut of NAFLD [89]. According to Nobili et al., in children with NAFLD, hepatic FXR and circulating FGF19 levels were lower than those in control subjects, decreasing FGFR4 signaling and promoting NAFLD [90]. Lou et al. reported that GPBAR1 activation by bile acids induces proinflammatory cytokine expression in Kupffer cells in a JNK-dependent manner and is correlated with suppression of CYP7A1 expression in hepatocytes, which contributes to bile acid feedback regulation [91].

To summarize, many preclinical and clinical studies have made significant progress uncovering bile acid metabolism and signaling and their contribution to NAFLD (Table 3). Alterations in the concentration and composition of the bile acid pool in NAFLD affect several metabolic pathways through FXR and GPBAR1 signaling molecules. Thus, restoring the bile acid pool will be a potential therapeutic strategy to treat NAFLD patients.

## 4. Microbiota-Derived Endogenous Ethanol

Clinical studies have shown that dysbiosis due to changes in microbiome composition in NASH patients who do not consume alcohol increases the level of ethanol in blood, suggesting that the gut microbiota might generate endogenous ethanol, which contributes to liver injury [16,92,93]. In children with NAFLD, blood ethanol levels were significantly higher than those in healthy children and were positively related to blood levels of insulin, leptin and triglycerides, which are the indicators of insulin resistance [92]. In addition to the elevated blood ethanol levels, children with NASH showed an increase in ethanol-producing bacteria in their gut microbiota [16]. These results suggest that gut microbiota-derived endogenous ethanol production might contribute to the development of NAFLD and progression toward NASH.

Preclinical and clinical studies have identified *E. coli*, *Enterobacteriaceae* spp. and *Klebsiella pneumoniae* as ethanol-producing bacteria that are relatively highly abundant in patients and mice with NAFLD [16,94]. High levels of microbiota-derived endogenous ethanol impair intestinal permeability via increased expression of inflammatory cytokines, resulting in increased portal endotoxemia and further triggering the inflammatory response [95]. In addition to ethanol, its metabolite, acetaldehyde, can also disrupt intestinal tight junctions and induce hepatic injury [96,97]. Ethanol metabolism can also generate reactive oxygen species and nitrogen species, causing oxidative stress and hepatocyte necrosis [98,99]. Ethanol can also increase the mRNA and protein expression of cytochrome P450 2E1 (CYP2E1), whose metabolism results in the release of free radicals, thereby contributing to mitochondrial dysfunction and possibly liver injury [99,100,101]. Endogenous ethanol inhibits the tricarboxylic acid cycle and increases the level of acetate, thus promoting triglyceride accumulation in hepatocytes [54]. These results indicate that elevated levels of endogenous ethanol may increase intestinal permeability, deteriorate liver inflammation and increase the levels of its metabolites, contributing to NAFLD pathogenesis.

In summary, studies have shown that a high abundance of endogenous ethanol-producing gut microbiota due to dysbiosis can induce NAFLD progression by directly and/or indirectly damaging the liver through an impaired intestinal barrier, increased portal endotoxemia and the release of free radicals (Table 4). Further analyses to identify ethanol-producing microbiota and their target signaling pathways need to be conducted to broaden our understanding of the impact of endogenous ethanol in NAFLD patients.

## 5. Microbiota-Derived Epigenetic Changes in Nonalcoholic Fatty Liver Disease

Epigenetics includes modifications of genes without changing the DNA sequence. These changes are generally caused by four mechanisms [102,103]. First, DNA can be methylated at cytosine or adenosine residues by DNA methyltransferases. Second, posttranslational modifications can occur at histone proteins, including acetylation, methylation, ubiquitination and phosphorylation. Third, changes in the expression profiles of enzymes regulating DNA methylation and histone acetylation can affect DNA and histone modifications. Last, posttranscriptional regulation by noncoding RNAs, such as microRNAs, can regulate gene expression and genome stability. These epigenetic modifications can alter chromatin structure, affecting transcriptional activation and repression and ultimately influencing fundamental physiological processes and inducing several diseases [104].

The molecular machinery responsible for regulating DNA and histone modifications is highly sensitive to metabolite availability. For example, histone acetyltransferases (HATs) use acetyl coenzyme A (acetyl-CoA), which is an essential metabolic intermediate of both catabolic and anabolic metabolism, to modify lysine groups on histone tails [105]. Another metabolite, *S*-adenosylmethionine (SAM), is utilized by histone and DNA methyltransferases as a vital methyl donor group for methylation. SAM is synthesized from the adenylation of methionine by methionine adenosyltransferase in the one-carbon cycle, which uses methyl groups derived from dietary folate in the folate cycle [106]. Dietary components, including choline and betaine, can also contribute to the synthesis of SAM. Choline, an essential nutrient for the structural integrity of cell membranes and lipid metabolism, provides one-carbon units for the synthesis of SAM in the one-carbon cycle [107,108]. Many studies have shown that diet-induced choline deficiency induces global DNA methylation [109,110,111]. This was recapitulated by choline-consuming bacteria in which intestinal microbial choline utilization reduced the bioavailability of choline and depleted methyl-donor metabolites, resulting in alterations in global DNA methylation and an increase in metabolic disease susceptibility [31].

Increasing evidence demonstrates the interplay between metabolites, gut microbes, epigenetics and disease [112]. This complexity is also shown in the progression of NAFLD, although the underlying mechanisms are under investigation. Kim et al. reported that high-fat or high-fructose diet-induced NAFLD in mice showed changes in DNA methylation patterns in specific liver genes compared to normal chow diet-fed mice, with hypomethylation at *Apoa4*, *Atp1a1*, *Kcnj16*, *Nfatc1* and *Plb1* genes and hypermethylation at *Fgfr1*, *Ptpn11*, *Shank2*, *Gria1* and *Col4a2*, all of which are associated with key metabolic pathways in the liver [113]. These changes were correlated with alterations in gut microbiome composition, especially with high elevation of *Akkermansia*, which was previously shown to be associated with metabolic disease [113]. Cordero et al. reported that methyl-donor supplementation induced changes in the methylation levels of a subset of genes involved in obesity development and lipid metabolism, including *Srebf2*, *Agpat3* and *Esr1*, in high-fat sucrose diet-fed rats, reducing lipid fat accumulation in the liver [114]. Zeybel et al. performed pyrosequencing assays to determine the DNA methylation status at specific CpGs within fibrosis-related genes in NAFLD patients with advanced and severe fibrosis and found different DNA methylation levels at specific CpGs of *PPARα*, *PPARδ*, *TGFβ*, *Collagen1A1* and *PDGFα* between the two groups. This suggests that DNA methylation levels at specific CpGs can be a useful tool to predict progression to liver fibrosis [115]. These findings were further supported by the Hotta group. In their study, CpG26 in the regulatory region of *PARVB* variant 1 was markedly hypomethylated and CpG99 in the regulatory region of *PNPLA3* was hypermethylated in patients with advanced NAFLD compared with those with mild NAFLD, all of which indicates the contribution of DNA methylation to fibrosis severity [116]. In addition to changes in DNA methylation, altered gut microbiota composition is associated with histone modifications, especially with histone acetylation, which plays a crucial role in the progression of NAFLD. Gut microbiota-derived SCFAs can act as histone deacetylase (HDAC) inhibitors and regulate gene expression changes [117]. HDACs remove acetyl groups from histone, resulting in chromatin closure and the prevention of gene expression. Chen et al. reported that deficiency of sirtuin 3 (SIRT3), a Class III HDAC, promotes NAFLD progression [118]. *SIRT3* knockout mice fed high-fat diet showed gut microbial dysbiosis with an increase in *Desulfovibrio* and *Oscillibacter* and a decrease in *Alloprevotella* and impaired intestinal permeability and inflammation and such liver injury was attenuated by supplementation with sodium butyrate [118]. Tian et al. showed that HDAC8 is associated with tumorigenesis in murine NAFLD-associated HCC models, where HDAC8 was upregulated by SREBP1 and its knockdown promoted insulin sensitivity and significantly reduced tumorigenicity [119].

There is increasing evidence that epigenetic modifications promote NAFLD progression by regulating the expression levels of genes involved in various host metabolic pathways, including lipid metabolism and inflammation (Table 5). To develop novel therapeutic strategies, further studies are required to understand how the gut microbiota and epigenetic mechanisms are related and how their association regulates NAFLD pathogenesis.

## 6. Conclusions

**Figure 1:**
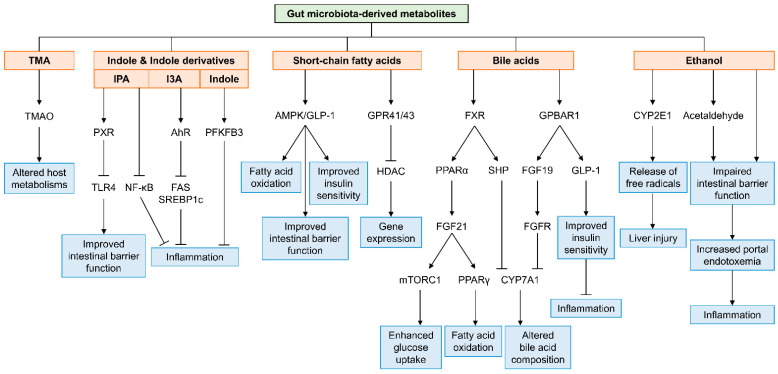
A schematic view of the mechanisms by which gut microbiota-derived metabolites are involved in the progression of nonalcoholic fatty liver disease. TMA, trimethylamine; TMAO, trimethylamine-N-oxide; IPA, indole-3-propionic acid; I3A, indole-3-acetic acid; PXR, pregnane X receptor; TLR4, Toll-like receptor 4; NF-κB, nuclear factor-kappa B; AhR, aryl hydrocarbon receptor; FAS, fatty acid synthase; SREBP1c, sterol regulatory element-binding protein 1c; PFKFB3, 6-phosphofructo-2-kinase/fructose-2,6-biphosphatase 3; AMPK, AMP-activated protein kinase; GLP-1, glucagon-like peptide-1; GPR41/43, G-protein coupled receptor 41 and 43; HDAC, histone deacetylase; FXR, farnesoid X receptor; PPARα, peroxisome proliferator-activated receptor α; FGF21, fibroblast growth factor 21; mTORC1, mammalian target of rapamycin complex 1; PPARγ, peroxisome proliferator-activated receptor γ; SHP, short heterodimer partner; GPBAR1, G-protein-coupled bile acid receptor 1; FGF19, fibroblast growth factor 19; FGFR, fibroblast growth factor receptor; CYP7A1, cytochrome P450 7A1; CYP2E1, cytochrome P450 2E1.

Preclinical and clinical studies have provided evidence that dysbiosis in gut microbiota is associated with the development of NAFLD. Changes in microbiota composition in the gut alter the profile of its metabolites, which drives the reprogramming of liver metabolism through direct and/or indirect mechanisms and ultimately leads to NAFLD progression. Among the metabolites derived from gut microbiota, in this review, we focused on five specific metabolite classes—TMA, indole and indole derivatives, SCFAs, bile acids and endogenous ethanol—and we discussed their contributions to NAFLD development in detail through preclinical and clinical studies. As summarized in Figure 1, these metabolites directly or indirectly interact with host signaling molecules in hepatocytes, immune cells and intestinal epithelial cells to modulate inflammation, intestinal barrier function, fatty acid oxidation and insulin sensitivity, contributing to liver damage. However, challenges remain to characterize the complex interplay between the gut microbiota, its metabolites and NAFLD progression. First, for the current metabolomics, false negatives (e.g., missing metabolite candidates) are usually a frequent and problematic occurrence, which can result in an incomplete set of microbial metabolites. Each metabolite has different stability and turnover rates inside cells [120]; thus, during the sampling procedure and instrumental analysis, bioactive metabolites can be significantly lost. To overcome such technical difficulties and the extensive diversity of metabolites, further technical advances in sample preparation and analysis methodologies and bioactive compound identification are required. Second, there are inconsistent and conflicting results among clinical studies. This may be caused by relatively small numbers of NAFLD patients and technical challenges. Additionally, this issue can result from the fact that current functional metabolomic studies mostly focus on identifying the role(s) of a single metabolite or an individual microbial taxon producing a specific metabolite of interest in NAFLD progression, which can generate biased results. The human metabolome consists of the interactions of metabolites produced from both host and microbiota. Therefore, studying a comprehensive signaling network of metabolites is needed to elucidate the dynamic interactions between metabolites and to obtain a comprehensive understanding of the interplay between the gut microbiota and host metabolism. In summary, effort to discover microbial metabolites has extended our knowledge to a great degree to comprehend the cellular and molecular mechanisms by which gut microbiota-derived metabolites are closely linked to NAFLD progression. With further advances in technologies, identifying new gut microbiota-derived metabolites and new host targets by understanding the dynamic interplay between gut microbiota and host metabolism will provide novel therapeutic strategies and diagnostic tools to improve liver diseases in patients.

## Figures and Tables

**Table 1:** Studies of gut microbiota-derived choline and tryptophan metabolites in nonalcoholic fatty liver disease.

Conditions		Treatment	Main Results	Ref.
Animal	High-fat diet	*cutC* gene knockout in *E. coli* MS-200	[↑] Global DNA methylation in liver [↓] TMAO, free fat acids in serum, liver triglyceride, adiposity	[31]
		Dietary TMAO	[↑] Fasting insulin levels, FOXO1, IL-6, CCL2 [↓] Glucose tolerance, IRS-2, PI3K, PKB, GYS2, GLUT2, IL-10	[34]
		Indole	[↓] Hepatic steatosis, inflammation	[38]
		Indole-3-propionic acid	[↑] *Firmicutes* to *Bacteroidetes* ratio, ZO-1, OCLN [↓] Plasma endotoxin, NF-κB signaling, TNF-α, IL-1β, IL-6, hepatic inflammation, liver injury, fibrogenic and collagen genes	[39]
		Indole-3-acetic acid	[↓] LPS-induced TNF-α, CCL2, IL-1β, FAS, SREBP1c	[40]
	Germ free	Indole	[↑] CLDN7, OCLN, ZO-1, CTNNB1, CDH1	[41]
	*Nr1i2* knockout (PXR deficient)	Indole-3-propionic acid	[↑] Intestinal permeability, TLR4 [↓] CLDN7, OCLN, ZO-1, CDH1	[42]
Human	NAFLD, women	Choline-deficient diet	[↑] *Gammaproteobacteria*, *Erysipelotrichi*, liver fat	[32]
	NAFLD	-	[↑] Circulating TMAO, NAFLD severity	[33]
	Obesity	-	[↑] BMI, liver fat, HOMA-IR [↓] Circulating indole	[38]

↑ indicates an increase in condition, ↓ indicates a decrease in condition. cutC, choline trimethylamine-lyase; TMAO, trimethylamine-N-oxide; FOXO1, forkhead box protein O1; CCL2, C-C motif chemokine 2; IL-6, interleukin 6; IRS-2, insulin receptor substrate 2; PI3K, phosphatidylinositol 3-kinase; PKB, protein kinase B; GYS2, liver-specific glycogen synthase; GLUT2, glucose transporter 2; IL-10, interleukin 10; ZO-1, zonula occluden 1; OCLN, occludin; NF-κB, nuclear factor-kappa B; LPS, lipopolysaccharide; TNF- α, tumor necrosis factor α; IL-1β, interleukin 1β; FAS, fatty acid synthase; SREBP1c, sterol regulatory element-binding protein 1c; CDH1, cadherin 1; CLDN7, claudin 7; CTNNB1, catenin beta 1; TLR4, Toll-like receptor 4; PXR, pregnane X receptor; NAFLD, nonalcoholic fatty liver disease; BMI, body mass index; HOMA-IR, homeostasis model assessment of insulin resistance.

**Table 2:** Studies of gut microbiota-related short-chain fatty acids in nonalcoholic fatty liver disease.

Conditions		Treatment	Main Results	Ref.
Animal	Polysaccharide- rich diet	*Gpr41* knockout	[↑] Intestinal transit rate, unabsorbed SCFAs [↓] Weight gain, total body fat, PYY	[60]
	Methionine- and choline-deficient diet	Acetate, propionate, butyrate	[↑] AMPK activation, CPT1a [↓] ALT, AST, lipid droplets, triglyceride, cholesterols, steatosis, inflammation, hepatic aggregation of macrophages	[62]
	High-fat diet	Acetate, propionate, butyrate	[↑] Fatty acid oxidation, insulin sensitivity, phospho-AMPK, phospho-ACC [↓] Steatosis, PPARγ expression and activity	[63]
		-	[↑] Propionate and butyrate in blood, SCFA-producing bacteria [↓] Endotoxin-secreting bacteria, proinflammatory cytokines	[61]
		-	[↑] Hepatic GLP-1, phospho-AMPK, phospho-ACC, IR, IRS-1 [↓] Steatosis	[67]
		-	[↑] *Christensenellaceae*, *Blautia*, *Lactobacillus*, ZO-1 [↓] Weight gain, endotoxins, TLR4, Myd88, CCL2, TNFα, IL-2, IL-6, IFNγ, triglyceride, cholesterol, ALT, AST	[69]
Human	NAFLD	-	[↑] Fecal acetate and propionate, *Fusobacterium*, *Prevotella*, *Eubacterium biforme*, T helper 17 cell/resting regulatory T cell ratio in peripheral blood mononuclear cell [↓] Resting regulatory T cells	[58]

↑ indicates an increase in condition, ↓ indicates a decrease in condition. SCFA, short-chain fatty acid; PYY, peptide-YY; AMPK, AMP-activated protein kinase; CPT1a, carnitine palmitoyltransferase 1a; ALT, alanine aminotransferase; AST, aspartate transaminase; ACC, acetyl-CoA carboxylase; PPARγ, peroxisome proliferator–activated receptor-γ; GLP-1, glucagon-like peptide 1; IR, insulin receptor; IRS-1, insulin receptor substrate 1; TLR4, Toll-like receptor 4; CCL2, C-C motif chemokine 2; TNFα, tumor necrosis factor α; IL-2, interleukin 2; IL-6, interleukin 6; IFNγ; interferon γ; NAFLD, nonalcoholic fatty liver disease.

**Table 3:** Studies of bile acids in nonalcoholic fatty liver disease.

Conditions		Treatment	Main Results	Ref.
Animal	High-fat diet	INT-767, dual FXR/GPBAR1 agonist	[↑] Energy expenditure [↓] GLP-1, hepatic steatosis, triglyceride, ALT, AST	[78]
	Methionine- and choline-deficient diet	*Gpbar1* knockout	[↑] NAS, TNFα, IL-6, M1 macrophage polarization, NLRP3 activation [↓] IL-4, IL-10	[79]
	High-fat diet	*Fxr* knockout	[↑] TβMCA, *Bacteroidetes* [↓] Hepatic steatosis, *Firmicutes*	[86]
Human	NAFLD	-	[↑] Serum primary and secondary bile acid concentrations, *Escherichia*, *Bilophila* [↓] CDCA, FXR- and FGFR4-mediated signaling	[89]
	NAFLD with fibrosis	-	[↑] Serum GCA correlated with *Bacteroidaceae*, fecal DCA correlated with *Lachnospiraceae*	[85]
	NAFLD, children	-	[↓] Hepatic FXR, circulating FGF19	[90]

↑ indicates an increase in condition, ↓ indicates a decrease in condition. FXR, farnesoid X receptor; GPBAR1, G-protein-coupled bile acid receptor 1; GLP-1, glucagon-like peptide-1; ALT, alanine aminotransferase; AST, aspartate transaminase; NAS, NAFLD activity score; TNFα, tumor necrosis factor α; IL-6, interleukin 6; NLRP3, NACHT, LRR and PYD domain-containing protein 3; IL-4, interleukin 4; IL-10, interleukin 10; TβMCA, tauro-β-muricholic acid; CDCA, chenodeoxycholic acid; FGFR4, fibroblast growth factor receptor 4; FGF19, fibroblast growth factor 19; GCA, glycocholic acid; DCA, deoxycholic acid; NAFLD, nonalcoholic fatty liver disease.

**Table 4:** Studies of microbiota-derived endogenous ethanol in nonalcoholic fatty liver disease.

Conditions		Main Results	Ref.
Animal	Normal diet with *Klebsiella pneumoniae* mutants with two different alcohol-producing abilities	[↑] Blood ethanol, steatosis, mitochondrial damage, ALT, AST, triglyceride	[101]
Human	NASH, children, obesity	[↑] Blood ethanol, *Proteobacteria*, *Enterobacteriaceae*, *Escherichia*	[16]
	NAFLD, children	[↑] Ethanol, insulin, leptin and triglyceride in blood	[92]
	NASH, women	[↑] Serum ethanol	[93]
	NASH	[↑] *K. pneumoniae*, feces ethanol	[94]
		[↑] ALDH1A/1B/1C/4/5/6 transcriptional activity, ALDH1/4 expression	[96]

↑ indicates an increase in condition. ALT, alanine aminotransferase; AST, aspartate transaminase; ALDH, alcohol dehydrogenase; NAFLD, nonalcoholic fatty liver disease; NASH, nonalcoholic steatohepatitis.

**Table 5:** Studies of microbiota-derived epigenetic changes in nonalcoholic fatty liver disease.

Conditions		Gene Profile	Main Results	Ref.
Animal	High-fat diet	Hypomethylation at *Apoa4*, *Atp1a1*, *Kcnj16*, *Plb1* Hypermethylation at *Fgfr1*, *Ptpn11*, *Shank2*, *Gria1*, *Col4a2*	[↑] Cholesterol, triglyceride, *Firmicutes* to *Bacteroidetes* ratio, *Oscillibacter*, *Parabacteroides*, *Clostridium*, *Anaerotruncus*, *Odoribacter*, *Desulfovibrio*, *Akkermansia*	[113]
		*Sirt3* knockout	[↑] *Desulfovibrio*, *Oscillibacter*, lipopolysaccharides in plasma and liver [↓] *Alloprevotella*, intestinal permeability, hepatic steatosis, inflammation	[118]
	High-fat sucrose diet with methyl-donor supplementation	Changes in methylation levels in *Srebf2*, *Agpat3*, *Esr1*	[↓] Liver fat accumulation	[114]
Human	NAFLD with different severity of fibrosis	Different methylation levels at fibrosis-related genes—*PPARα*, *PPARδ*, *TGFβ*, *Collagen1A1*, *PDGFα*—according to fibrosis severity	[↑] Hepatocyte ballooning and portal inflammation in patients with advanced fibrosis NAFLD patients	[115]
		Hypomethylation at CpG26 of *PARVB* variant 1 Hypermethylation at CpG99 of *PNPLA3* in patients with advanced NAFLD	[↓] *PNPLA3* mRNA levels in patients with advanced NAFLD	[116]

↑ indicates an increase in condition, ↓ indicates a decrease in condition. NAFLD, nonalcoholic fatty liver disease.

## Data Availability

Data are contained within the article.
